# Perioperative PD-1 inhibitor plus chemotherapy versus chemotherapy alone in gastric cancer with PAN metastasis undergoing D2 gastrectomy and PAND: a single-center retrospective cohort study

**DOI:** 10.3389/fimmu.2026.1835041

**Published:** 2026-08-12

**Authors:** Zeyao Ye, Weixing Wu, Pengfei Yu, Ling Huang, Xiangliu Chen, Yang Cao, Tengjiao Chai, Yian Du

**Affiliations:** 1 Department of Gastric Surgery, Zhejiang Cancer Hospital, Hangzhou Institute of Medicine (HIM), Chinese Academy of Sciences, Hangzhou, Zhejiang, China; 2 Postgraduate Training Base Alliance of Wenzhou Medical University (Zhejiang Cancer Hospital), Hangzhou, Zhejiang, China

**Keywords:** gastric cancer, immunotherapy, neoadjuvant chemotherapy, para-aortic lymph nodes, pathological response

## Abstract

**Objectives:**

Research on the efficacy of perioperative immunotherapy for gastric cancer with para-aortic lymph node (PAN) metastasis remains limited. This study aimed to compare the safety and efficacy of chemotherapy plus programmed cell death protein-1 (PD-1) inhibitor versus chemotherapy alone in locally advanced GC patients with PAN metastasis.

**Methods:**

We retrospectively reviewed patients with gastric cancer and limited No.16a2/b1 lymph node metastasis who received perioperative systemic therapy combined with D2 gastrectomy plus PAN dissection (PAND) between January 2018 and February 2024. Patients were divided into Group A (chemotherapy+PD-1 inhibitor, n=62) and Group B (chemotherapy alone, n=71). Pathological response, survival outcomes, and safety were evaluated.

**Results:**

All patients in Group A achieved R0 resection, compared with 98.6% in Group B. Pathological outcomes were significantly better in Group A, with higher rates of pathological complete response (pCR; 37.1% vs. 4.2%, P<0.001) and major pathological response (MPR; 58.1% vs. 22.5%, P<0.001) compared to Group B. In Group A, patients with programmed death ligand-1 (PD-L1) combined positive score (CPS) ≥5 had higher pCR rates. The 3-year overall survival (OS) rate was also higher in Group A (64.5% vs. 42.3%). The median OS for Group B was 33.2 months, while Group A was not reached (P = 0.023). Adverse event incidences were comparable between groups, with no treatment-related deaths.

**Conclusion:**

Perioperative immunochemotherapy significantly enhances pathological response and survival in GC patients with PAN metastasis, with manageable safety. This regimen represents a promising therapeutic strategy for this patient population, necessitating long-term follow-up to confirm durable benefits.

## 1 Introduction

Gastric cancer (GC) is the fifth most common malignancy worldwide and the fifth leading cause of cancer-related deaths, imposing a substantial global disease burden (1). Surgical resection with curative intent remains the cornerstone of treatment for early-stage and locally advanced disease, where the adequacy of resection margins and the completeness of lymph node dissection are pivotal determinants of long-term survival (2). Notably, metastasis to the para-aortic lymph nodes (PAN, station 16) signifies a particularly aggressive disease subgroup. The International Union Against Cancer (UICC) TNM staging system categorizes No.16 lymph nodes involvement as distant metastasis, consistent with the associated poor prognosis, which is reflected in 5-year overall survival (OS) rate of merely 16%–21% (3). However, contrary to the conventional view that distant metastasis precludes curative surgery, growing clinical evidence suggests that an aggressive surgical approach incorporating PAN dissection (PAND) may confer a survival advantage in a selected subset of patients with limited PAN metastasis (4).

In recent decades, diverse treatment strategies for GC have been explored to improve patient survival. Among these strategies, perioperative chemotherapy has gained increasing recognition as a key component of multimodal management (5). Some studies have demonstrated that perioperative chemotherapy significantly improves survival in advanced GC compared to adjuvant chemotherapy alone (5, 6). Particularly, for those with suspected PAN metastasis but without other non-curative factors such as visceral organ involvement, neoadjuvant chemotherapy represents a promising therapeutic option. Patients exhibiting a favorable response to chemotherapy may subsequently undergo D2 + PAND resection, which can increase the likelihood of R0 resection and potentially improve long-term survival (7). This approach is supported by the Japanese Gastric Cancer Treatment Guidelines (6th edition), which recommend neoadjuvant chemotherapy followed by PAND for patients with isolated metastasis to the No. 16a2/b1 lymph nodes (8). However, due to the intrinsic heterogeneity of GC and interpatient variability, standard neoadjuvant chemotherapy regimens have shown limited effectiveness in achieving higher pathological response rates and substantial survival benefits. Therefore, exploring more effective treatment modalities to improve the prognosis of such patients remains crucial.

In recent years, immunotherapy has emerged as a transformative modality in various solid tumors, with programmed cell death protein-1 (PD-1) and programmed cell death ligand 1 (PD-L1) serving as key immune checkpoint inhibitors (ICIs), offering substantial clinical benefits for GC patients (9, 10). PD-1 is prominently expressed on immune cells, and its interaction with PD-L1, often upregulated on tumor cells, promotes immune tolerance within the tumor microenvironment. Specifically, PD-1 inhibitors prevent the interaction between the PD-1 receptor and PD-L1, thereby triggering a strong immune response against the tumor and exerting anti-tumor effects (11). Studies have shown that anti-PD-1 therapy is effective in GC patients, especially when combined with cytotoxic chemotherapy in the neoadjuvant setting, leading to significantly improved pathological response rates (12–14). Furthermore, the use of PD-L1 expression levels as a biomarker for predicting the efficacy of immunotherapy has gained traction. For example, the CheckMate-649 trial demonstrated that patients with a PD-L1 combined positive score (CPS) ≥5 experienced significantly greater survival benefits compared to both the overall population and those with PD-L1 CPS ≥1 (10). Despite these advances, the role of immunotherapy remains inadequately explored in the specific subgroup of GC patients with PAN metastases. Moreover, the safety and efficacy of ICIs in the neoadjuvant setting for these specific patients remain uncertain (15, 16).

GC patients are often diagnosed at an advanced stage, with retrospective studies reporting that approximately 10% to 40% present with PAN metastasis (17–19). However, perioperative clinical studies specifically targeting this patient population remain limited and often yield inconclusive results. To fill this critical gap, we conducted a retrospective study in patients with locally advanced gastric cancer (LAGC) and isolated No. 16a2/b1 lymph node metastasis, comparing perioperative chemotherapy alone versus chemotherapy combined with PD-1 inhibitors to evaluate treatment efficacy. We also aim to assess the impact on surgical outcomes and the safety of these treatments, ultimately exploring perioperative treatment strategies for GC with PAN metastasis, with the hope of providing valuable practical guidance for clinical practice.

## 2 Methods

### 2.1 Patients

Clinicopathologic data were retrospectively reviewed for patients with GC and isolated No.16a2/b1 PAN metastasis who received perioperative treatment at Zhejiang Cancer Hospital (Hangzhou, China) between January 2018 and February 2024.

Patients were enrolled according to the following criteria: (1) age 18–75 years; (2) histologically confirmed gastric adenocarcinoma; (3) abdominal contrast-enhanced computed tomography (CT) indicating enlarged PANs in the 16a2 and/or 16b1 regions, with a short-axis diameter ≥10 mm; (4) received perioperative chemotherapy with or without PD-1 inhibitor, along with radical gastrectomy and PAND; (5) Eastern Cooperative Oncology Group (ECOG) performance status of 0–1; and (6) adequate organ function to tolerate chemotherapy, immunotherapy, and surgery. The exclusion criteria included: (1) presence of distant metastases confirmed by enhanced CT, PET-CT, or intraoperative findings; (2) concurrent malignancies or refractory autoimmune disease; (3) history of partial or segmental gastrectomy; (4) chronic use of corticosteroids or immunosuppressive agents; (5) lack of pathologically confirmed gastric adenocarcinoma; (6) missing postoperative pathological results; and (7) completely missing follow-up data resulting in undetermined survival status and time.

**Figure 1:**
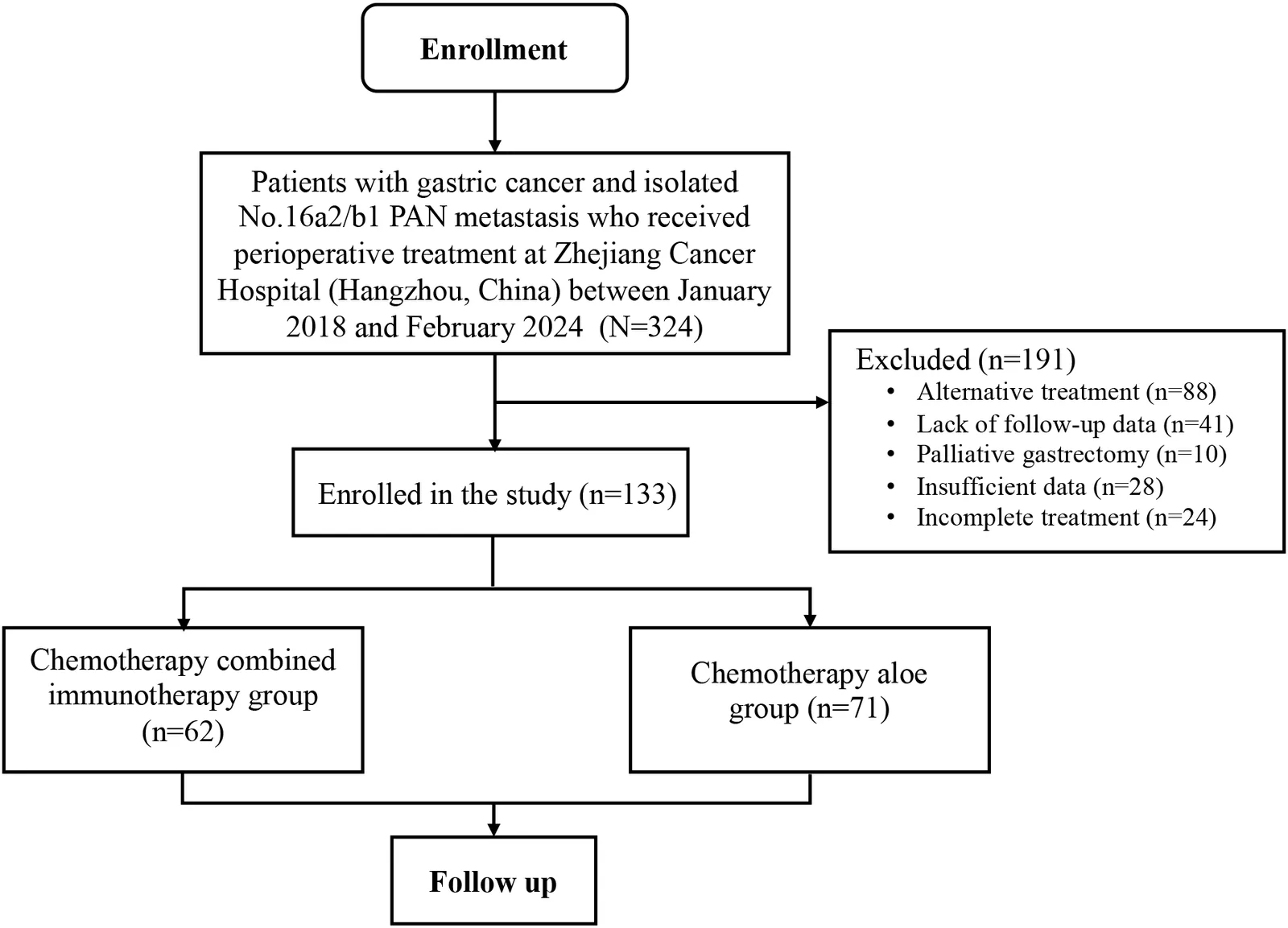
Study flowchart of patient selection.

A total of 324 patients were initially identified. Of these, 191 patients were excluded due to alternative treatment (n=88), lack of follow-up data (n=41), palliative gastrectomy (n=10), insufficient data (n=28), or incomplete treatment (n=24), leaving 133 cases for analysis (**Figure 1**). Patients receiving chemotherapy combined with immunotherapy were classified to Group A (n=62), and those receiving chemotherapy alone were classified to Group B (n=71).

This study was approved by the Ethics Committee of Zhejiang Cancer Hospital (Approval No. IRB-2025-844) and conducted in accordance with the Declaration of Helsinki. Given the retrospective nature of this study, the Ethics Committee granted exemption from obtaining informed consent.

### 2.2 Treatment

#### 2.2.1 Perioperative chemotherapy and immunotherapy

Patients in group A received a combination of chemotherapy and PD-1 inhibitor, while patients in group B received chemotherapy alone. Preoperative treatment consisted of 2–4 cycles, and postoperative treatment involved 4–6 cycles, with each cycle lasting 21 days. Chemotherapy regimens were selected based on physician discretion and included the following: SOX regimen (S-1 [60 mg twice daily from day 1 to day 14] and oxaliplatin [130 mg/m² administered on day 1, repeated every 21 days]); XELOX regimen (capecitabine [1000 mg/m², twice daily from day 1 to day 14] and oxaliplatin [130 mg/m² on day 1, repeated every 21 days]); AS regimen (S-1 [60 mg twice daily from day 1 to day 14] and nab-paclitaxel [125 mg/m² administered on days 1 and 8, repeated every 21 days]); PS regimen (S-1 [40–60 mg twice daily from day 1 to day 14] and paclitaxel [80 mg/m² on days 1, 8, and 15, repeated every 21 days]); DCF regimen (docetaxel [75 mg/m² on day 1 via intravenous infusion], cisplatin [75 mg/m² on day 1 via intravenous infusion], and 5-fluorouracil [750–1000 mg/m² via continuous intravenous infusion for 96 hours starting on day 1, repeated every 21 days]). The neoadjuvant immunotherapy regimen involved PD-1 inhibitors, including 200 mg of serplulimab or 200 mg of sintilimab, administered intravenously once every 21 days.

#### 2.2.2 Surgery

**Figure 2:**
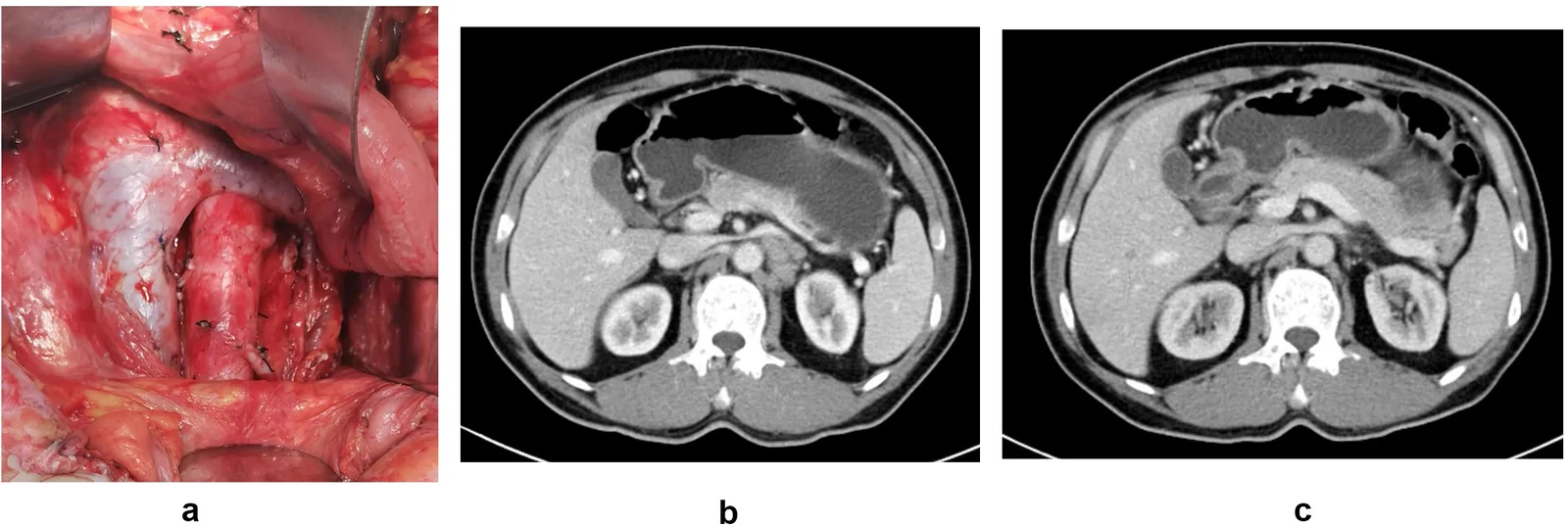
Representative images from patients with gastric cancer and PAN metastasis undergoing D2 gastrectomy and PAND. **(a)** Surgical field following PAND. **(b)** Pre-treatment radiographic assessment. **(c)** Post-treatment radiographic assessment.

All patients underwent open D2 radical gastrectomy combined with PAND according to the Japanese Gastric Cancer Treatment Guidelines (**Figure 2a**) (8, 20). Depending on the tumor location, patients received distal or total gastrectomy, followed by Roux-en-Y esophagojejunostomy, Billroth II gastrojejunostomy, or Billroth I gastroduodenostomy.

### 2.3 Endpoints and evaluation

The primary endpoint was the pathological complete response (pCR) rate. Secondary endpoints included safety, major pathological response (MPR), R0 resection rate, OS, and the pathological status of PAN.

Adverse events (AEs) were graded according to the Common Terminology Criteria for Adverse Events (CTCAE), version 3.0 (21). Postoperative complications were graded using the Clavien-Dindo classification system (22). A CT scan is performed every 6–8 weeks to evaluate the tumor (**Figures 2b, c**) (23). Residual tumor status was categorized as R0 (no residual tumor), R1 (microscopic residual tumor), or R2 (macroscopic residual tumor), in accordance with the residual tumor (R) classification system (24). Pathological tumor regression in resected specimens was assessed by two experienced pathologists using the Becker criteria and categorized according to the Tumor Regression Grading (TRG) system as follows: TRG1a, no residual tumor cells; TRG1b, <10% residual tumor cells; TRG2, 10-50% residual tumor cells; and TRG3, >50% residual tumor cells (25). The pCR (TRG1a) was defined as the absence of viable tumor cells in the specimen. The MPR (TRG1a/b) was defined as less than 10% residual tumor cells after preoperative treatment. Tumor downstaging was assessed by comparing clinical and pathological staging.

In Group A, PD-L1 expression was assessed using immunohistochemistry (Clone 22C3, Dako) and quantified with the CPS, calculated as the number of PD-L1-positive cells (tumor cells, lymphocytes, and macrophages) divided by the total number of viable tumor cells, multiplied by 100. A CPS≥1 was considered PD-L1-positive.

### 2.4 Follow-up

After treatment, patients were followed up every three months for the first two years and every six months from the third to the fifth year. Follow-up was conducted via telephone interviews and outpatient visits, including routine physical examinations, imaging assessments, and blood tests (including tumor markers). OS was calculated from diagnosis to death or the last follow-up. The final follow-up date was June 8, 2026.

### 2.5 Statistical analysis

Categorical variables were expressed as frequencies and percentages, and group comparisons were conducted using either the chi-square test or Fisher’s exact test, as appropriate. Continuous variables were expressed as mean ± standard deviation or median (interquartile range, IQR), and comparisons were made using Student’s t-test or the Mann-Whitney U test, depending on data distribution. Survival curves were plotted using the Kaplan-Meier method and compared using the log-rank test. To identify independent prognostic factors, variables with a *P*-value < 0.10 in univariate analysis were included in the multivariate Cox proportional hazards model using stepwise selection. Results were presented as hazard ratios (HRs) and 95% confidence intervals (CIs). A two-tailed *P*-value of <0.05 was considered statistically significant. All statistical analyses were performed using SPSS software (version 26.0; SPSS Inc., Chicago, IL, USA).

## 3 Results

### 3.1 Patient characteristics

Among the 133 patients included, 95 (71.4%) were male, with a median age of 66 years (range: 28–75 years). Histologically, 84.2% of the patients had poorly differentiated adenocarcinoma. The tumor location was primarily in the stomach (74.4%), while 25.6% of the tumors were located at the gastroesophageal junction. Postoperative pathological findings revealed a significantly lower proportion of PAN-positive cases in Group A compared with Group B (14.5% vs. 29.6%, *P* = 0.038). Additionally, vascular tumor emboli were less common in Group A than in Group B (30.6% vs. 54.9%, *P* = 0.005), and perineural invasion was observed less frequently in Group A (29.0% vs. 53.5%, *P* = 0.004). Regarding pathological response, a significantly higher proportion of patients in Group A achieved complete tumor regression and nodal clearance. Specifically, the rates of ypT0 were 41.9% in Group A versus 9.9% in Group B, and yN0 rates were 50.0% versus 14.1%, respectively (both *P* < 0.001). Detailed clinicopathologic characteristics are summarized in **Tables 1**, **2**.

**Table 1:** Baseline characteristics.

Variable	Group A (n=62)	%	Group B (n=71)	%	*P*-value
Gender					0.510
Male	46	74.2%	49	69.0%	
Female	16	25.8%	22	31.0%	
Age					0.390
<60	20	32.3%	28	39.4%	
≥60	42	67.7%	43	60.6%	
ECOG					0.520
0	54	87.1%	59	83.1%	
1	8	12.9%	12	16.9%	
cT-stage					0.762
≤T2	2	3.2%	3	4.2%	
>T2	60	96.8%	68	95.8%	
cN-stage					0.291
≤N2	8	12.9%	14	19.7%	
N3	54	87.1%	57	80.3%	
Tumor location					0.581
Upper	14	22.6%	20	28.2%	
Middle	20	32.3%	25	35.2%	
Lower	28	45.2%	26	36.6%	
Differentiation					0.184
Well/moderately	7	11.3%	14	19.7%	
Poorly/undifferentiated	55	88.7%	57	80.3%	
Signet-ring cells					0.728
Positive	11	17.7%	11	15.5%	
Negative	51	82.3%	60	84.5%	
ASA					0.590
I	2	3.2%	5	7.0%	
II	54	87.1%	58	81.7%	
III	6	9.7%	8	11.3%	
Serum CEA (U/mL)					0.067
Normal	46	74.2%	42	59.2%	
>5	16	25.8%	29	40.8%	
Serum CA125 (U/mL)					0.905
Normal	52	83.9%	59	83.1%	
>35	10	16.1%	12	16.9%	
Serum CA199 (U/mL)					0.405
Normal	46	74.2%	48	67.6%	
>37	16	25.8%	23	32.4%	

**Table 2:** Pathological characteristics.

Variable	Group A (n=62)	%	Group B (n=71)	%	*P*-value
PAN					0.038
Positive	9	14.5%	21	29.6%	
Negative	53	85.5%	50	70.4%	
Vascular tumor embolus					0.005
Negative	43	69.4%	32	45.1%	
Positive	19	30.6	39	54.9%	
Nerve infiltration					0.004
Negative	44	71.0%	33	46.5%	
Positive	18	29.0%	38	53.5%	
Serum CEA (U/mL)					0.582
Normal	55	88.7%	65	91.5%	
>5	7	11.3%	6	8.5%	
Serum CA125 (U/mL)					0.280
Normal	30	48.4%	41	57.7%	
>35	32	51.6%	30	42.3%	
Serum CA199 (U/mL)					0.582
Normal	55	88.7%	65	91.5%	
>37	7	11.3%	6	8.5%	
ypT-stage					<0.001
T0	26	41.9%	7	9.9%	
≥T1	36	58.1%	64	90.1%	
ypN-stage					<0.001
N0	31	50.0%	10	14.1%	
≥N1	31	50.0%	61	85.9%	

### 3.2 Perioperative treatment

Both groups received a median of three neoadjuvant treatment cycles and five adjuvant treatment cycles. Following neoadjuvant therapy, all patients underwent D2 radical gastrectomy with PAND. The R0 resection rate was 100% in Group A (62/62) and 98.6% in Group B (70/71), with no statistically significant difference between the two groups (*P* = 0.348). In Group B, two patients additionally required splenectomy due to tumor invasion.

Despite similar median values, the estimated blood loss in Group B was significantly higher than that in Group A (100 mL [IQR:100–200 mL] vs. 100 mL [IQR: 50–100 mL], *P* < 0.001). Furthermore, Group A had more lymph nodes retrieved compared to Group B (40.5 [IQR: 17-76] vs. 32 [IQR: 14-75], *P* = 0.005). Additionally, the number of No.16a2/b1 lymph nodes retrieved was greater in Group A than in Group B (6 [IQR: 3-9] vs.3 [IQR: 1-6], *P* = 0.003). No significant differences were found in hospitalization days (16 days [IQR: 12–20 days] vs. 17 days [IQR: 11–21 days], *P* = 0.495), nasogastric tube retention days (4.56 ± 1.15 days vs. 4.63 ± 1.28 days, *P* = 0.213), or first flatus days (3.2 ± 1.19 days vs. 3.5 ± 1.08 days, *P* = 0.484) between Group A and Group B. Within 30 days post-surgery, 45 patients (33.8%) experienced surgery-related complications. No significant differences were found between Group A and Group B in overall complication rates (35.5% vs. 32.4%, *P* = 0.848) or severe complication rates (4.8% vs. 2.8%, *P* = 0.877). The most common postoperative complications in both groups were hypoproteinemia and anemia. In Group A, there were three instances of severe complications, including dyspnea, wound dehiscence, and heart failure; in Group B, two severe complications occurred, including dyspnea and pleural effusion. No treatment-related deaths were reported in either group (**Table 3**).

**Table 3:** Comparison of surgical characteristics and postoperative recovery.

Surgical Outcomes	Group A (n=62)	Group B (n=71)	*P*-value
**Operation time**, **min (mean ± SD)**	243.15 ± 54.82	229.96 ± 58.55	0.184
**Estimated blood loss**, **mL (median**, **IQR)**	100 (50-100)	100 (100-200)	**<0.001**
**Retrieved lymph nodes**, **n (median**, **IQR)**	40.5 (17-76)	32 (14-75)	**0.005**
**Retrieved No.16a2/b1 lymph nodes**	6 (3-9)	3 (1-6)	**0.003**
**Hospitalization days**	16 (12-20)	17 (11-21)	0.495
**Nasogastric tube retention days**	4.56 ± 1.15	4.63 ± 1.28	0.213
**First flatus days**	3.2 ± 1.19	3.5 ± 1.08	0.484
**Days to first liquid diet**	3.39 ± 1.16	3.38 ± 1.25	0.210
Radical resection [n (%)]			0.348
R0	62 (100.0%)	70 (98.6%)	
R1	0 (0.0%)	1 (1.4%)	
Tumor resection			0.970
Distal	26 (41.9%)	30 (42.3%)	
Total	36 (58.1%)	41 (57.7%)	
Reconstruction method [n (%)]			0.382
Billroth I	0 (0.0%)	3 (4.2%)	
Billroth II	25 (40.3%)	27 (38.0%)	
Roux-en-Y esophagojejunostomy	37 (59.7%)	41 (57.8%)	
Clavien-dindo classification			
Grade II			
Hypoproteinemia	4	3	
Anemia	3	4	
lymphatic leakage	3	2	
Pancreatic fistula	2	2	
Pneumonia	2	2	
Aminotransferase increased	1	2	
Intestinal obstruction	1	1	
Urinary Retention	1	1	
Gastroparesis	0	2	
Diarrhea	1	1	
Intra-abdominal infection	1	0	
Atrial fibrillation	0	1	
Grade III			
Dyspnea	1	1	
Pleural effusion	0	1	
Wound dehiscence	1	0	
Grade IV			
heart failure	1	0	
**Total complication rate [n (%)]**	22 (35.5%)	23 (32.4%)	0.848
**Severe complication rate [n (%)]**	3 (4.8%)	2 (2.8%)	0.877

Bold values indicate statistically significant differences (P < 0.05).

### 3.3 Tumor regression and survival

Postoperatively, tumor response was evaluated based on Becker criteria for pathological regression of resected specimens. In the entire cohort, 26 patients (19.5%) achieved pCR, with 23 patients (37.1%) in Group A and 3 patients (4.2%) in Group B. The difference between the groups was statistically significant (*P* < 0.001). The MPR rate was significantly higher in Group A compared to Group B (58.1% vs. 22.5%; *P* < 0.001; **Table 4**). A sensitivity analysis excluding the two patients who received the triplet chemotherapy (both in Group B) yielded consistent results, with Group A maintaining significantly higher rates of pCR (37.1% vs. 4.3%; *P* < 0.001) and MPR (58.1% vs. 23.2%; *P* < 0.001) (**Supplementary Table 1**). Additionally, preoperative PD-L1 expression data were available for 50 patients in Group A, among whom 35 (70.0%) were PD-L1 positive (CPS≥1). The pCR rates were 37.1% and 20.0% in PD-L1-positive and PD-L1-negative patients, respectively (*P* = 0.328), and the MPR rates were 60.0% and 40.0% in the respective groups (*P* = 0.193). Further analysis indicated that patients with a CPS ≥5 had significantly higher pCR (46.1% vs. 16.7%; *P* = 0.035) and MPR (73.1% vs. 33.3%; *P* = 0.010) rates than those with CPS <5 (**Table 5**).

**Table 4:** Pathological evaluation of response.

Variable	Group A (n=62)	%	Group B (n=71)	%	*P*-value
**TRG**					**<0.001**
0	23	37.1%	3	4.2%	
1	13	21.0%	13	18.3%	
2	22	35.5%	47	66.2%	
3	4	6.5%	8	11.3%	
**pCR**	23	37.1%	3	4.2%	**<0.001**
**MPR**	36	58.1%	16	22.5%	**<0.001**

Bold values indicate statistically significant differences (P < 0.05).

**Table 5:** Associations between the expression of PD-L1 and pathological response.

Variable	CPS < 1 (n = 15)	CPS ≥ 1 (n = 35)	*P*-value	CPS < 5 (n = 24)	CPS ≥ 5 (n = 26)	*P*-value
TRG			0.466			**0.023**
1a	3 (20.0%)	13(37.1%)		4 (16.7%)	12 (46.2%)	
1b	3 (20.0%)	8 (22.9%)		4 (16.7%)	7 (26.9%)	
2	8 (53.3%)	11 (31.4%)		14 (58.3%)	5 (19.2%)	
3	1 (6.7%)	3 (8.6%)		2 (8.3%)	2 (7.7%)	
pCR	3 (20.0%)	13 (37.1%)	0.328	4 (16.7%)	12 (46.1%)	**0.035**
MPR	6 (40.0%)	21 (60.0%)	0.193	8 (33.3%)	19 (73.1%)	**0.010**

Bold values indicate statistically significant differences (P < 0.05).

**Figure 3:**
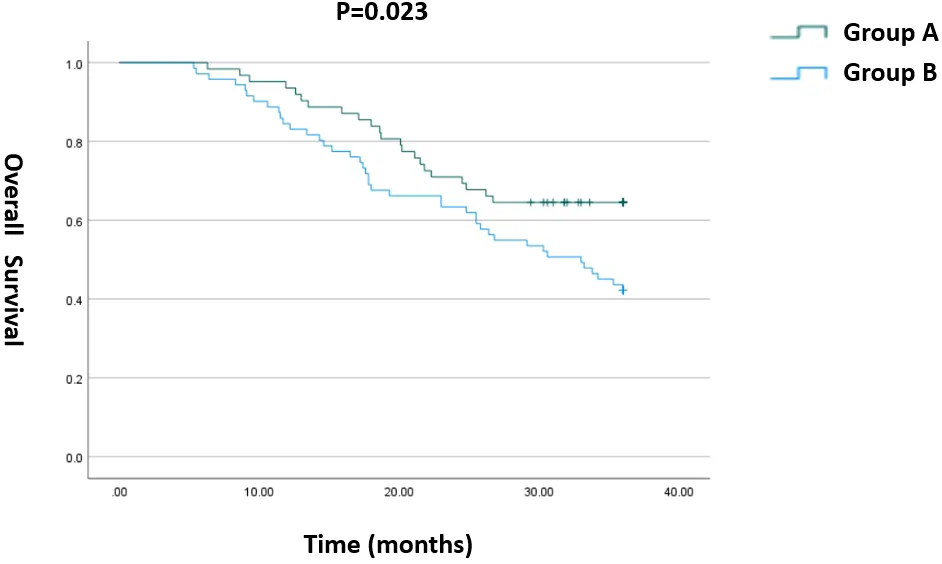
Kaplan-Meier estimate of overall survival between Group A and Group B.

**Figure 4:**
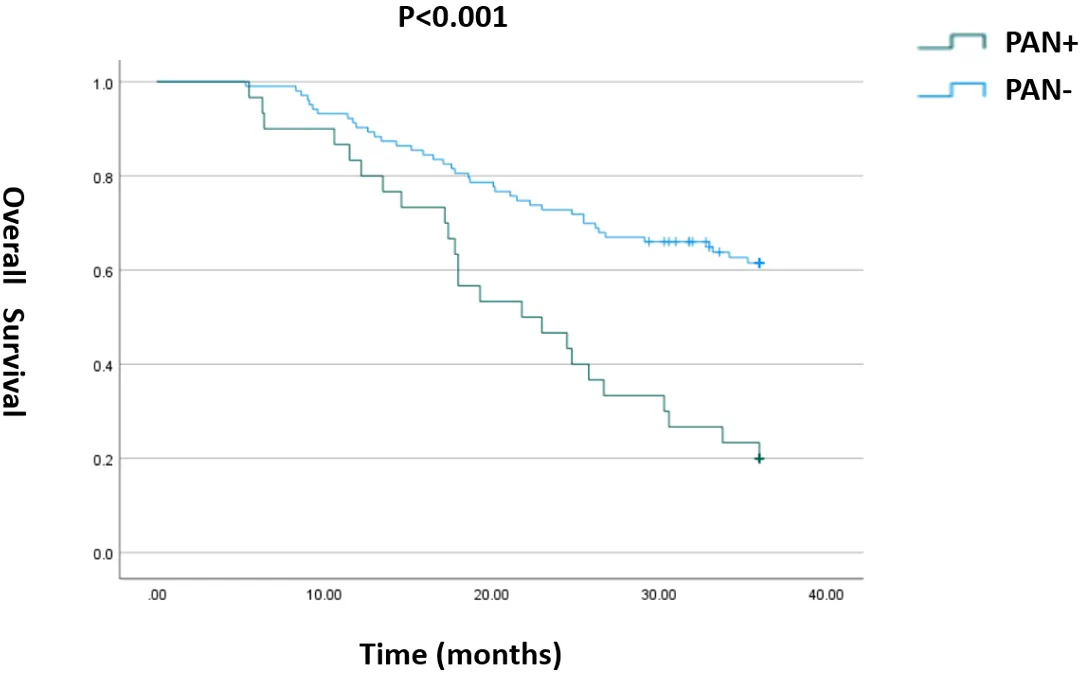
Kaplan-Meier estimate of overall survival between patients with PAN positive and those with PAN negative.

**Figure 5:**
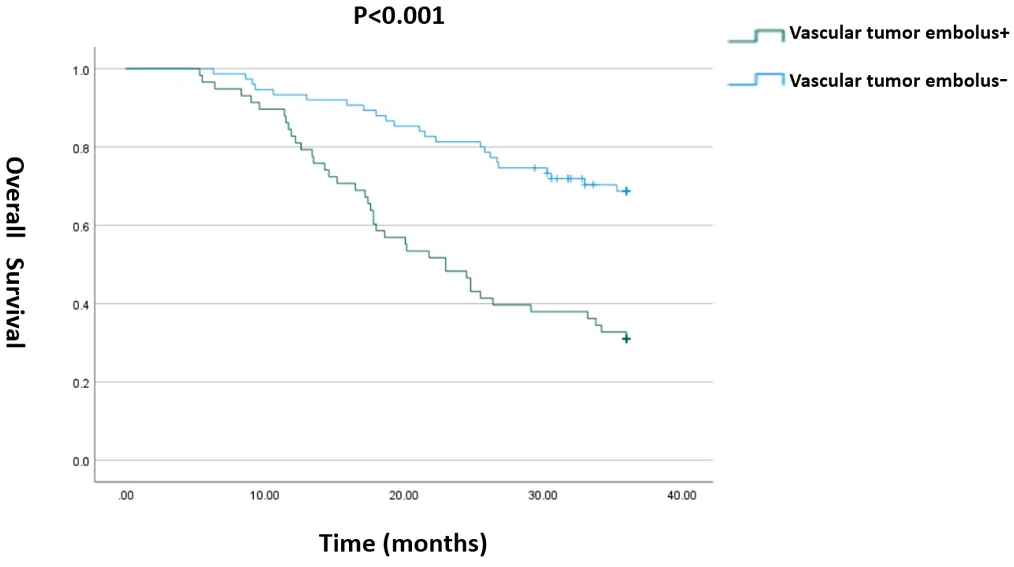
Kaplan-Meier estimate of overall survival between patients with vascular tumor embolus positive and those with vascular tumor embolus negative.

All discharged patients were followed up, with a median follow-up period of 33.2 months (range: 6–72 months). At data cutoff, the median OS for Group B was 33.0 months, while the median OS for Group A was not reached (NR; *P* = 0.023; **Figure 3**). OS rates in Group A were consistently higher than in Group B at 1-year (93.5% vs. 84.5%), 2-year (71.0% vs. 63.4%), and 3-year (64.5% vs. 42.3%). Sensitivity analysis excluding the two patients who received the triplet chemotherapy confirmed this results (*P* = 0.023; **Supplementary Figure 1**). In the entire cohort, 30 patients had pathological positivity in the 16th lymph node group postoperatively (9 in the Group A and 21 in the Group B; *P* = 0.038), and their prognosis was significantly worse compared to patients with negative pathology (**Figure 4**). Additionally, patients with vascular tumor embolus positive had significantly worse OS than those with vascular tumor embolus negative (*P* < 0.001; **Figure 5**).

In the univariate analysis, pCR achievement was significantly associated with improved OS (HR = 0.286, 95% CI: 0.115–0.714, *P* = 0.007). However, after adjusting for other significant clinicopathological variables in the multivariate model, the prognostic significance of pCR was attenuated and it was no longer identified as an independent predictor of survival (*P* = 0.353). Instead, the multivariate analysis identified signet ring cell carcinoma (HR = 1.917, 95% CI: 1.032–3.162, *P* = 0.043), lymphovascular invasion (HR = 2.084, 95% CI: 1.173–2.121, *P* = 0.012), positive No.16 lymph nodes (HR = 2.146, 95% CI: 1.241–3.711, *P* = 0.006), and higher pN stage (HR = 2.303, 95% CI: 1.252–4.236, *P* = 0.007) as independent risk factors for worse OS (**Table 6**). The identification of these independent risk factors remained unchanged in the sensitivity analysis excluding the two patients who received the triplet chemotherapy (**Supplementary Table 2**).

**Table 6:** Univariate and stepwise multivariate Cox regression analysis of overall survival.

Variable	Univariate analysis		Multivariate analysis	
	HR (95% CI)	P-value	HR (95% CI)	P-value
Age(years)	0.996(0.593–1.671)	0.986	–	–
Male gender	1.297(0.763–2.206)	0.337	–	–
ECOG PS	0.961(0.474–1.946)	0.912	–	–
Differentiation	1.014(0.516–1.993)	0.968	–	–
Signet ring cell	2.001(1.436–2.789)	<0.001	1.917(1.032–3.162)	0.043
Lymphovascular invasion	3.162(1.888–5.294)	<0.001	2.084 (1.173–2.121)	0.012
Nerve invasiont	2.072(1.261–3.405)	0.004	1.155 (0.629–4.844)	0.642
pCR achievement	0.286(0.115–0.714)	0.007	0.620 (0.226–1.701)	0.353
No.16 lymph node positive	2.890(1.731–4.823)	<0.001	2.146 (1.241–3.711)	0.006
pN	3.146(1.860–5.323)	<0.001	2.303(1.252–4.236)	0.007

### 3.4 Safety assessment

Safety profile is displayed in **Table 7**. A total of 122 patients (91.7%) experienced at least one AE of any grade during perioperative treatment, with no significant difference in incidence between Group A (56/62; 90.3%) and Group B (66/71; 92.9%) (*P* = 0.582). Grade 3 or 4 adverse events were reported in 13 patients (21.0%) of Group A and in 17 patients (23.9%) of Group B, with no statistically significant difference between two groups (*P* = 0.682). The most common hematologic toxicities in the entire cohort were leukopenia, neutropenia, and anemia, while the most frequent non-hematologic toxicities were elevated transaminases, vomiting, and pneumonia. Two patients in Group A developed grade 3 hypothyroidism, which was successfully managed with thyroid hormone therapy. All adverse events were well-controlled with appropriate treatments.

**Table 7:** Treatment-related adverse events during perioperative systemic therapy.

Safety	Group A (n, %)		Group B (n, %)	
	Any grade	Grade3-4	Any grade	Grade3-4
**Total**	56 (90.3)	13 (21.0)	66 (92.9)	17 (23.9)
Hematological				
Anemia	29 (46.8)	3 (4.8)	33 (46.5)	4 (5.6)
Leucopenia	34 (54.8)	3 (4.8)	37 (52.1)	5 (7.0)
Neutropenia	25 (40.3)	2 (3.2)	28 (39.4)	3 (4.2)
Thrombocytopenia	19 (30.6)	2 (3.2)	19 (38.0)	3 (4.2)
Non-hematological				
Nausea/vomiting	28 (45.2)	1 (1.6)	30 (42.2)	1 (1.4)
Elevated ALT/AST	18 (29.0)	2 (3.2)	16 (22.5)	3 (4.2)
Dermatitis	12 (19.4)	0 (0.0)	11 (15.5)	1 (1.4)
Peripheral sensory neuropathy	10 (16.1)	0 (0.0)	9 (12.7)	0 (0.0)
Diarrhea	10 (16.1)	0 (0.0)	11 (15.5)	0 (0.0)
Pneumonia	5 (8.1)	0 (0.0)	5 (7.0)	0 (0.0)
Fatigue	5 (8.1)	0 (0.0)	9 (12.7)	0 (0.0)
Elevated creatinine	4 (6.5)	0 (0.0)	3 (4.2)	0 (0.0)
Constipation	2 (3.2)	0 (0.0)	4 (5.6)	0 (0.0)
Dermatitis	1 (1.6)	0 (0.0)	1 (1.4)	0 (0.0)

## 4 Discussion

In this study, we evaluated the efficacy and safety of chemotherapy combined with PD-1 inhibitors compared to chemotherapy alone as perioperative treatment for LAGC patients with limited No.16a2/b1 lymph node metastasis. Our findings demonstrated that patients receiving the perioperative combination therapy experienced higher pCR and downstaging rates than those receiving chemotherapy alone, contributing to significantly improved survival outcomes. Notably, patients with a CPS≥5 exhibited significantly enhanced tumor regression rates. Through multivariate Cox regression analysis, we identified several clinicopathological factors that are independently associated with poorer survival outcomes, including the presence of signet ring cell carcinoma, lymphovascular invasion, positive No.16 lymph nodes, and higher pN stage. Importantly, the addition of PD-1 inhibitors did not lead to an increased risk of surgical complications or adverse events, with a generally manageable safety profile.

Historically, extended lymphadenectomy (D2+PAND) was considered the standard surgical approach for LAGC patients, before emerging evidence challenged its utility. The JCOG9501 trial showed that prophylactic PAND did not confer survival benefit and was associated with higher perioperative complication rates compared to D2 surgery alone (26). However, therapeutic PAND may benefit selected LAGC patients with isolated No.16a2/b1 metastasis following neoadjuvant chemotherapy, as evidenced by improved prognoses in several clinical studies (27–29). The clinical utility of neoadjuvant chemotherapy in GC remains constrained by suboptimal pathological response rate and unsatisfactory long-term outcomes, necessitating the need for more effective perioperative strategies. Immunotherapy represents a significant breakthrough in advanced GC management, introducing new opportunities for neoadjuvant treatment. Previous evidence suggested that immunotherapy may synergistically enhance the antitumor activity of chemotherapy (30). The landmark phase III CheckMate 649 trial demonstrated that first-line nivolumab combined with chemotherapy significantly improved median OS compared to chemotherapy alone (11.1 to 13.1 months; HR: 0.71, 98.4% CI: 0.59-0.86; *P* < 0.001) (10). Building upon the established efficacy in advanced disease, ICIs emerged as a promising modality for perioperative treatment. Although the phase III KEYNOTE-585 trial did not meet its primary endpoint of event-free survival (EFS), the addition of pembrolizumab to cisplatin-based chemotherapy resulted in significantly increase in pCR rate (12.9% vs. 2.0%; *P* < 0.00001) (31). Our previous work also demonstrated enhanced pathological responses with perioperative SOX plus PD-1 inhibitors in patients with LAGC (14). However, studies specifically targeting LAGC patients with PAN metastasis are limited. Our current study demonstrated that perioperative chemotherapy combined with PD-1 inhibitors significantly improved pathological responses and reduced the proportion of postoperative PAN positivity in LAGC patients with No.16a2/b1 lymph node metastasis. Additionally, we observed that conventional binary PD-L1 classification (positive vs. negative) failed to discriminate pCR and MPR, whereas stratification by CPS thresholds (CPS ≥5) uncovered a significant difference in pathological responses. This association is consistent with established biological mechanisms, wherein higher CPS scores reflect increased target density for immune checkpoint blockade and greater pre-existing T-cell infiltration (32). Emerging evidence from the CheckMate-649 (10) and GEMSTONE-303 (33) trials support our findings, suggesting that a CPS threshold of ≥5 may be the minimum requirement for clinically meaningful immune activation in gastrica malignancies.

In addition to enhancing pathological response, perioperative immunochemotherapy provided significant survival benefit over chemotherapy alone for LAGC patients (median OS: NR vs. 33.0 months; *P* = 0.023), with consistently higher OS rates observed during long-term follow-up. However, whether the pathological regression advantage translates into improved survival for LAGC patients receiving perioperative treatment remains uncertain. While Chen et al. reported that GC patients achieving pCR following neoadjuvant therapy exhibited superior survival outcomes (34), our study did not demonstrate a statistically significant association between pCR and OS (*P* = 0.353). This discrepancy may be attributable to the limited sample size or to the confounding effects of other dominant biological factors and tumor subtypes that drive survival outcomes more strongly than pathological response alone. Furthermore, patients with pathologically negative PAN had better survival compared to those positive. Notably, the rate of postoperative pathologically confirmed PAN positivity was more than two-fold higher in the chemotherapy group than in the immunochemotherapy group (29.6% vs. 14.5%), suggesting improved regional disease control with PD-1 inhibitor-based regimens. Despite aggressive postoperative intervention, patients with positive PAN had significantly worse prognoses than those negative, suggesting the critical role of No. 16a2/b1 nodes as a conduit for cancer dissemination and a sanctuary site for immune evasion within the tumor microenvironment. These findings underscore the importance of comprehensive pathological assessment and risk stratification in guiding postoperative management and follow-up strategies for patients with PAN-positive GC.

Given the potential surgical challenges posed by perioperative chemotherapy and immunotherapy, evaluating short-term surgical outcomes is critical. The JCOG0405 trial, which adopted the S-1 plus cisplatin regimen, reported key surgical metrics for D2+PAND, including a median operative time of 350 minutes, median blood loss of 847 mL, a pancreatic fistula rate of 20% (with no grade III-IV complications), and an anastomotic leak rate of 6.1%, with no treatment-related mortality. The R0 resection rate was 88%, and the response rate was 65% (35). In terms of neoadjuvant immunotherapy, a Japanese multicenter, single-arm phase I clinical trial enrolled 31 LAGC patients who received two cycles of nivolumab monotherapy before surgery, with 30 patients eventually undergoing surgery. Postoperatively, five patients (16.1%) experienced grade III complications, including three cases of anastomotic leaks and two cases of pancreatic fistulas (16). However, limited data exist on the short-term outcomes of D2+PAND surgery following neoadjuvant chemotherapy combined with immunotherapy. Our findings indicated that chemotherapy combined with immunotherapy did not increase perioperative complication rates, supporting the feasibility of this combination regimen. Additionally, compared to chemotherapy alone, patients receiving combination therapy underwent more extensive lymph node dissection during surgery, significantly enhancing the accuracy of pathological staging. Moreover, intraoperative blood loss was notably lower in immunochemotherapy group, potentially improving surgical safety and reflecting reduced vascularity or inflammation in the operative field.

A meta-analysis evaluating perioperative safety found no significant difference in the incidence of grade 3–4 adverse events in GC patients receiving neoadjuvant immunochemotherapy and those receiving chemotherapy alone (20.6% vs. 25.7%, *P* = 0.380) (36). Consistent with this, our study showed that adding PD-1 inhibitors to chemotherapy did not significantly increase the incidence of adverse events compared to chemotherapy alone, indicating that the toxicity of PD-1 inhibitors is tolerable. However, potential immune-related adverse events (irAEs) remain a critical concern that warrants close monitoring. Previous studies have shown that organ-specific irAEs typically involve the endocrine system and skin, followed by the gastrointestinal tract and lungs, with most events being grade 1–2 in severity (17, 18). In our study, five patients (6.7%) in the combination therapy group experienced irAEs, including two cases of grade 3 hypothyroidism requiring hormone replacement therapy. As ICIs are increasingly incorporated into the treatment landscape for LAGC, early recognition and timely management of irAEs are essential to optimize patient safety and compliance (37).

The remarkable pathological response rates observed in our study can be attributed to the synergistic mechanisms between chemotherapy and PD-1 inhibitors. In the context of locally advanced gastric cancer with para-aortic lymph node (PAN) metastasis, this combination exerts a dual-pronged attack on the tumor microenvironment (TME).Firstly, chemotherapy acts as an “immunological primer.” Cytotoxic agents such as oxaliplatin and taxanes not only induce direct tumor cell lysis but also promote the release of tumor-associated antigens (TAAs) and damage-associated molecular patterns (DAMPs). This process, known as Immunogenic Cell Death (ICD), enhances the visibility of tumor cells to the host immune system by facilitating dendritic cell maturation and antigen presentation. In the specific setting of PAN metastasis—a region often characterized by immunosuppressive stromal barriers—this antigen release is crucial for breaking immune tolerance. Secondly, PD-1 inhibitors function as an “immune checkpoint releaser.” By blocking the PD-1/PD-L1 interaction, these agents reinvigorate exhausted CD8+ T cells within the tumor microenvironment. Notably, our subgroup analysis indicated that patients with a CPS ≥5 derived the most significant benefit. This aligns with the mechanistic understanding that a pre-existing immune infiltrate (often reflected by higher CPS) is necessary for ICI efficacy. The chemotherapy-induced antigen release likely expands the repertoire of tumor-reactive T cells, which are then sustained and activated by the PD-1 blockade. Furthermore, emerging evidence suggests that chemotherapy may also deplete immunosuppressive cell populations, such as regulatory T cells (Tregs) or myeloid-derived suppressor cells (MDSCs), within the metastatic lymph nodes. This “remodeling” of the PAN metastatic niche from a “cold” to a “hot” tumor microenvironment provides a compelling biological rationale for the high downstaging rates observed in our cohort. This mechanistic synergy underscores why the combination regimen is particularly potent in eradicating metastatic lymph nodes, a critical determinant of survival in this patient population.”

Regarding the baseline characteristics and selection bias: We acknowledge the non-randomized nature of this retrospective study. To assess the robustness of our baseline data and address potential selection bias, we conducted a 1:1 Propensity Score Matching (PSM) analysis. The results demonstrated that the Standardized Mean Differences (SMD) for all covariates were less than 20% both before and after matching. Furthermore, the P-values for the matched groups were all greater than 0.05, indicating no statistically significant differences in baseline characteristics between the two groups. Given this high degree of balance, we believe the original cohort adequately represents the population, and thus the main analysis was based on the full dataset without further PSM stratification.

Regarding treatment heterogeneity: We acknowledge that this study involved heterogeneous treatment regimens, including SOX, XELOX, and a few cases of triplet therapy (DCF/TPF). However, the vast majority of patients received either SOX or XELOX, which are considered standard and interchangeable backbones in gastric cancer treatment. Notably, the triplet regimens were administered to only 2 patients in the chemotherapy-alone group, minimizing their potential confounding effect on the comparison. This was directly confirmed by our sensitivity analyses, which demonstrated that excluding these two patients did not alter the significant improvements in pathological response and overall survival. Furthermore, both sintilimab and serplulimab used in this study are anti-PD-1 antibodies with comparable pharmacodynamics. This heterogeneity reflects the real-world clinical practice and is unlikely to affect the overall conclusion regarding the efficacy of PD-1 inhibitors.

This study has inherent limitations. First, it is a single-center retrospective analysis, which may introduce selection bias and influence the generalizability of the results. Second, the limited sample size and inherent treatment heterogeneity in this real-world cohort precluded meaningful standardization or subgroup classification of neoadjuvant regimens or treatment cycles. Future large-scale prospective studies with uniform treatment protocols are needed to identify the most effective regimen and clarify the contribution of each treatment component. Third, although excluding patients with incomplete treatment data was methodologically necessary, this may reduce the real-world applicability of our safety analysis, as early treatment discontinuation often reflects clinical practice realities. Fourth, although preliminary survival benefits are encouraging, confirmation of sustained therapeutic benefit will require longer follow-up and more mature survival data. Fifth, the subgroup analysis based on PD-L1 CPS was exploratory. Although we observed a trend toward higher pCR rates in patients with CPS ≥5, the relatively small sample size, especially in the CPS <5 subgroup, limits the statistical power of this analysis. Furthermore, this limited sample size and relatively immature follow-up data precluded us from conducting reliable survival analyses for these PD-L1 subgroups. Therefore, these findings should be interpreted with caution and validated in larger prospective cohorts. Finally, this study did not assess additional biomarkers that may predict treatment outcomes, such as microsatellite instability (MSI) or tumor mutational burden (TMB). Similarly, PD-L1 testing covered only 80.6% of patients in Group A. These biomarkers were largely unavailable for most patients in the real-world cohort, primarily attributable to significant patient out-of-pocket expenses, and insufficient residual tissue for complementary biomarker assays after initial diagnostics. Despite these limitations, our findings provide clinically relevant insights that may inform the design of future research on the perioperative management of LAGC patients with PAN metastasis.

## 5 Conclusion

In summary, integrating PD-1 inhibitors with standard chemotherapy significantly improved pathological response rates and OS among GC patients with PAN metastases, particularly in those with a PD-L1 CPS ≥5, while maintaining a manageable safety profile comparable to chemotherapy alone. These findings support the use of ICIs in combination with chemotherapy as a promising strategy to improve long-term outcomes in this high-risk population. Nonetheless, prospective studies are essential to validate the durability of survival benefits, and well-designed randomized controlled trials are warranted to establish the optimal perioperative strategy for GC patients with PAN metastasis.

## Data Availability

The original contributions presented in the study are included in the article/**Supplementary Material**. Further inquiries can be directed to the corresponding author.
