# Supplementary material for: Perioperative PD-1 inhibitor plus chemotherapy versus chemotherapy alone in gastric cancer with PAN metastasis undergoing D2 gastrectomy and PAND: a single-center retrospective cohort study
Source: Front Immunol. 2026 Aug 12;17:1835041. doi: 10.3389/fimmu.2026.1835041 (PMC13506907; doi:10.3389/fimmu.2026.1835041)
Supplement: Supplementary file 1 [file Table1.docx]

Supplementary Material

# 1 Supplementary Figure

**Supplementary Figure 1.** Kaplan-Meier survival curves for overall survival in the sensitivity analysis cohort. The analysis excludes two patients in Group B who received the triplet chemotherapy.

# 2 Supplementary Tables

**Supplementary Table 1**. Comparison of pathological response between Group A and Group B after excluding two patients who received the triplet chemotherapy.

| **Variable** | **Group A (n=62)** | **%** | **Group B (n=69)** | **%** | ***P*-value** |
| --- | --- | --- | --- | --- | --- |
| **TRG** |  |  |  |  | **<0.001** |
| 0 | 23 | 37.1% | 3 | 4.3% |  |
| 1 | 13 | 21.0% | 13 | 18.8% |  |
| 2 | 22 | 35.5% | 45 | 65.2% |  |
| 3 | 4 | 6.5% | 8 | 11.6% |  |
| **pCR** | 23 | 37.1% | 3 | 4.3% | **<0.001** |
| **MPR** | 36 | 58.1% | 16 | 23.2% | **<0.001** |

**Supplementary Table 2.** Univariate and multivariate Cox regression analyses for overall survival by excluding two patients who received the triplet chemotherapy.

| **Variable** | Univariate analysis | | Multivariate analysis | |
| --- | --- | --- | --- | --- |
|  | **HR (95% CI)** | **P-value** | **HR (95% CI)** | ***P*-value** |
| Age(years) | 0.992(0.589–1.669) | 0.976 | - | - |
| Male gender | 1.303(0.765–2.221) | 0.330 | - | - |
| ECOG PS | 0.964(0.476–1.955) | 0.920 | - | - |
| Differentiation | 1.120(0.552–2.270) | 0.754 | - | - |
| Signet ring cell | 2.005(1.440–2.796) | <0.001 | 1.970(1.102–3.353) | 0.041 |
| Lymphovascular invasion | 3.057(1.822–5.131) | <0.001 | 2.148 (1.188–4.017) | 0.011 |
| Nerve invasiont | 1.955(1.210–3.289) | 0.007 | 1.145 (0.679–1.926) | 0.760 |
| pCR achievement | 0.286(0.115–0.715) | 0.007 | 0.642 (0.218–2.701) | 0.585 |
| No.16 lymph node positive | 2.926(1.748–4.897) | <0.001 | 2.241 (1.204–3.979) | 0.008 |
| pN | 3.236(1.896–5.524) | <0.001 | 2.400(1.290–4.468) | 0.006 |
